# Pain Acceptance Predicts Expansive Outlooks on the Future in Older Chronic Pain Patients

**DOI:** 10.1093/geroni/igaa057.1395

**Published:** 2020-12-16

**Authors:** Gillian Fennell, Abby Pui Wang Yip, M Cary Reid, Susan Enguidanos, Elizabeth Zelinski, Corinna Loeckenhoff

**Affiliations:** 1 University of Southern California, Los Angeles, California, United States; 2 Cornell University, Ithaca, New York, United States; 3 Weill Cornell Medical College, New York City, New York, United States

## Abstract

As chronic conditions continue to rise in the US, associated pain symptoms are rising as well, affecting 65% of those 65 and older. In an attempt to help patients lessen the burdensome physical/psychological effects of chronic pain, researchers have investigated the effectiveness of therapeutic interventions with pain acceptance-based models yielding the most promising effect sizes. However, these interventions do not explicitly account for how patients perceive their future. Qualitative work has shown that chronic pain patients with positive and expansive views of their futures report fewer pain-related anxiety and depression symptoms, and are more likely to engage in long-term (and often more effective) treatment regiments. This study aims to investigate whether pain acceptance scores predict future time perspective to enhance treatment effects of chronic pain interventions. Multivariate linear regression analyses were conducted with a sample of 148 non-cancer patients age 45 and older with chronic pain, i.e. pain lasting three or more months. Pain duration, neuroticism, sex, race, income, and age were included in the model to explore potential mediating or moderating effects. A significant positive association was found between pain acceptance and future time perspective (r=.42, p<.001, r2=.17). Additionally, with the inclusion of all covariates, our model significantly explained 24.1% of the variance in future time perspective in the sample, F(7,132)=5.99, p<.001. With an established association between these two psychological constructs, strategies to bolster future time perspective can easily be integrated into pain acceptance interventions for older chronic pain patients, hopefully pushing effect sizes past the ‘moderate’ level.

